# Anxiety and depression with neurogenesis defects in exchange protein directly activated by cAMP 2-deficient mice are ameliorated by a selective serotonin reuptake inhibitor, Prozac

**DOI:** 10.1038/tp.2016.129

**Published:** 2016-09-06

**Authors:** L Zhou, S L Ma, P K K Yeung, Y H Wong, K W K Tsim, K F So, L C W Lam, S K Chung

**Affiliations:** 1 School of Biomedical Sciences, The University of Hong Kong, Pokfulam, Hong Kong SAR, China; 2 Department of Psychiatry, The Chinese University of Hong Kong, Shatin, Hong Kong SAR, China; 3 State Key Laboratory of Pharmaceutical Biotechnology, The University of Hong Kong, Pokfulam, Hong Kong SAR, China; 4 Division of Life Science and the Biotechnology Research Institute, Hong Kong University of Science and Technology, Clear Water Bay, Hong Kong SAR, China; 5 State Key Laboratory of Molecular Neuroscience, Hong Kong University of Science and Technology, Clear Water Bay, Hong Kong SAR, China; 6 Division of Life Science and Center for Chinese Medicine, Hong Kong University of Science and Technology, Clear Water Bay, Clear Water Bay, Hong Kong SAR, China; 7 Research Center of Heart, Brain, Hormone and Healthy Aging, The University of Hong Kong, Pokfulam, Hong Kong SAR, China; 8 State Key Laboratory of Brain and Cognitive Science, The University of Hong Kong, Pokfulam, Hong Kong SAR, China; 9 Department of Ophthalmology, The University of Hong Kong, Pokfulam, Hong Kong SAR, China

## Abstract

Intracellular cAMP and serotonin are important modulators of anxiety and depression. Fluoxetine, a selective serotonin reuptake inhibitor (SSRI) also known as Prozac, is widely used against depression, potentially by activating cAMP response element-binding protein (CREB) and increasing brain-derived neurotrophic factor (BDNF) through protein kinase A (PKA). However, the role of Epac1 and Epac2 (Rap guanine nucleotide exchange factors, RAPGEF3 and RAPGEF4, respectively) as potential downstream targets of SSRI/cAMP in mood regulations is not yet clear. Here, we investigated the phenotypes of Epac1 (Epac1^−/−^) or Epac2 (Epac2^−/−^) knockout mice by comparing them with their wild-type counterparts. Surprisingly, Epac2^−/−^ mice exhibited a wide range of mood disorders, including anxiety and depression with learning and memory deficits in contextual and cued fear-conditioning tests without affecting Epac1 expression or PKA activity. Interestingly, rs17746510, one of the three single-nucleotide polymorphisms (SNPs) in *RAPGEF4* associated with cognitive decline in Chinese Alzheimer's disease (AD) patients, was significantly correlated with apathy and mood disturbance, whereas no significant association was observed between *RAPGEF3* SNPs and the risk of AD or neuropsychiatric inventory scores. To further determine the detailed role of Epac2 in SSRI/serotonin/cAMP-involved mood disorders, we treated Epac2^−/−^ mice with a SSRI, Prozac. The alteration in open field behavior and impaired hippocampal cell proliferation in Epac2^−/−^ mice were alleviated by Prozac. Taken together, Epac2 gene polymorphism is a putative risk factor for mood disorders in AD patients in part by affecting the hippocampal neurogenesis.

## Introduction

Overlapping cerebral regions, circuits and cellular signaling pathways underpin comorbid cognitive decline and emotional disorders.^1^ Therefore, understanding the common molecular and cellular mechanisms underlying the progression of cognitive decline and concomitant affective traits, such as anxiety, apathy and depression, is critical for developing potential therapies of both cognitive impairment and mood disorders. Recently, it is reported that anxiety is associated with the accumulation of *in vivo* biomarkers t-tau and Aβ_42_ in the cerebrospinal fluid of patients with mild cognitive impairment.^2^ Correspondingly, anxiety and neuronal alteration in the hippocampus are found in a young Alzheimer's disease (AD) mouse model without amyloid deposition.^3^ The early involvement of the emotionality factor may reflect the underlying AD pathology and provide new strategies for an earlier diagnosis.

The functional significance of adult hippocampal neurogenesis has been demonstrated in learning and memory,^4^ emotional behaviors^5^ and neurodegenerative disorders, such as AD.^6^ Efficacy of selective serotonin reuptake inhibitors (SSRIs), such as fluoxetine, involved the upregulation of hippocampal neurogenesis.^7^ Fluoxetine and rolipram (selective PDE_4_ inhibitor) exert their antidepressant-like and neurogenic effects by increasing the cAMP level, and subsequently activating cAMP response element-binding protein (CREB).^8, 9^ Thus far, the effects of antidepressants on CREB are mostly analyzed with regard to the cAMP-dependent protein kinase A (PKA) pathway and its target brain-derived neurotrophic factor (BDNF).^10^ However, it is not clear whether the PKA/CREB pathway directly targets the proliferating progenitor cells or indirectly increases neurogenesis by promoting survival of polysialylated-neural cell adhesion molecule-positive immature neurons.^11^ Besides, Epac has been found to mediate cAMP signaling independent of PKA; therefore, the mechanistic role of the cAMP signaling pathway in cognitive and affective functions has to be re-evaluated. The two isoforms of Epac, Epac1 and Epac2, directly bind to cAMP and activate the Ras-like small GTPase Rap1/2.^12^ Epac1 is ubiquitously expressed, whereas Epac2 is relatively restricted in the brain and the adrenal glands.^13^ Recent microarray data have shown that Epac2 is highly expressed in the amygdala and the hippocampus of human brains, which are the regions responsible for emotional reaction and learning.^14^ Altered expression and activation of Rap1 and Epac2 in the hippocampus are shown in a post-mortem study on depressed suicide victims.^15^ The selective PKA inhibitor (Rp-8-Br-cAMP) increases cAMP levels in the hippocampus of rats, and reduces their behavioral despairs in the forced swimming test,^16^ suggesting that Epac may exert its antidepressive effects through pathways downstream to the cAMP signal. Supporting such notion, stimulation with the Epac analog shows beneficial effects on hippocampus-dependent memory functions in mice.^17, 18^ However, it is not clear which isoform of Epac is predominantly contributive to the antidepressant effect and improves cognitive function.

In the present study, Epac wild-type (WT), Epac1^−/−^, Epac2^−/−^ and Epac1;Epac2 double knockout (Epac1^−/−^;2^−/−^) mice were used to investigate the roles of Epac in anxiety and depressive disorders, as well as cognitive deficits. Further, to study the impact of Epac mutation in human disease, we examined several single-nucleotide polymorphisms (SNPs) in *RAPGEF3* and *RAPGEF4* (gene encoding Epac1 and Epac2, respectively) of Chinese AD patients, and performed the Neuropsychiatric Inventory (NPI) scoring.

## Materials and methods

### Animals

All animal experiments were performed according to the guidelines set forth by the Committee on the Use of Live Animals in Teaching and Research at The University of Hong Kong. Mice were housed with a 12-h light/dark cycle (L 0700 to 1900 hours) and *ad libitum* access to food and water. Targeted deletion of Epac1 was achieved by homologous recombination in a process described in detail previously.^19^ Epac2^−/−^ mice were kindly provided by Professor Susumu Seino's group at Kobe University Graduate School of Medicine.^20^ Epac1^−/−^ and Epac2^−/−^ mice were backcrossed to the C57BL/6 for at least 10 generations. Epac1^−/−^;2^−/−^ mice were generated by cross-breeding with Epac1^−/−^ and Epac2^−/−^ mice. C57BL/6 mice were used as WT control. Eight- to twelve-week male WT, Epac1^−/−^, Epac2^−/−^ and Epac1^−/−^;2^−/−^ mice were used in all experiments, unless otherwise stated.

### Behavioral tests

#### Open field test

Open field test is a well-established behavioral assessment to the locomotor activity and anxiety related behavior.^21^ The open field was a transparent (26 cm length × 26 cm width × 40 cm height) box with equivalent mouse bedding. The 10 cm × 10 cm square in the center of the box was defined as center arena. Four mice were detected in individual boxes at the same time and cardboards were placed in between to avoid disturbance. Mice were allowed to move freely in the open field for 10 min or 1 h. Their movements were video tracked and analyzed by EthoVision 3.0 (Noldus, Wageningen, The Netherlands). Total distance moved and time spent in center area were calculated accordingly.

#### Forced swimming test

A transparent cylinder (25 cm height × 10 cm diameter) was filled up with water up to a height of 10 cm, as described earlier.^22^ Fresh water was equivalent to the room temperature and changed for individual mouse. The camera was oriented for horizontal viewing. A sheet of cardboard was placed in-between the two cylinders so that the mice could not see each other. The movement of mice was recorded by software EthoVision 3.0 and divided into 3 kinds: immobility, mobility and strong mobility. Duration of immobility time was recorded in total 6 min.

#### Sucrose preference test

Mice were individually housed and given two bottles of liquid: a bottle with plain water and the other one with 1% sucrose. Bottles were reversed every 12 h. After 3 days of habituation, all bottles were removed at 1000 hours. After 12 h water deprivation, mice were given access to bottles of water and sucrose, in the reversed location. The bottles were reversed again 1 h later and weighed on the following morning. Sucrose preference was expressed as (Δ weight-sucrose)/(Δ weight-sucrose+Δ weight-water) × 100.^5^

#### Circadian rhythm assessment

This experiment was performed in Hong Kong University of Science and Technology, with 12:12 light/dark cycle (L 0600 to 1800 hours). Mice were housed individually in cages with running wheels linked to detectors.^23^ Day 1–10 was the habituation period. On day 11, the dark cycle was advanced by 6 h (L 0000 to 1200 hours) and mice were allowed to re-entrain for another 10 days. Time spent for adapting to the new light dark cycle was recorded. Average wheel running activity in light phase and dark phase was calculated before light dark shift (day 8 - day 10), and after light dark shift (day 18 - day 20). Water and food were provided *ad libitum*. The wheel running activity was recorded and analyzed using the Clocklab software (Actimetrics, Wilmette, IL, USA).

#### Pavlovian fear conditioning

This experiment was performed as previously described.^24^ Briefly, on day 1, mouse received a training session of 13.5 min in conditioning chamber (25 cm × 25 cm × 25 cm), where the mouse could freely explore in the chamber. The training consisted of a habituation period of 6 min, followed by 3 times of paired presentations of tone (conditional stimuli) and foot shock (unconditional stimuli). The foot shock (0.5 mA) was applied to the floor grid of the chamber during the final 2s of the tone (30s, 4 Hz, 80 dB). There was 2-min rest every time after the tone-shock pairing. The chambers were wiped and cleaned with 70% ethanol between training sessions. On day 2, fear conditioning to the context was assessed by returning the mice to the same chamber for 8 min, without tone or shock stimuli. On day 3, fear to the tone conditional stimuli was assessed in novel chamber with explicit cue as day 1, in the absence of foot shock. The chambers were cleaned with diluted creamy soap to remove olfactory debris. The contextual and cued tests scheme was applied again to the mice 1 week after the training. EthoVision XT7 (Noldus) detection system was used and the videos were saved for later behavioral analysis. A complete suppression of spontaneous locomotion and movements was counted as freezing behavior.

#### Morris Water Maze

Morris Water Maze with automatic tracking system was employed for assessing the spatial learning and memory as described before.^21^ All the experimental procedures were performed within the light cycle (0900 to 1300 hours). The mice were tested in three blocks of training in 9 consecutive days: visible platform training, hidden platform training and probe test. In trainings with platform, the mice were allowed to swim to reach the platform for a maximum period of 1min. Otherwise they were guided to the platform by the experimenter. In the visible platform test, no external cue was applied. Mice were trained to reach the flag-cued visible platform for 2 days with 4 trials per day. The platform location and starting position varied for each trial. In the hidden platform training, external cues were hanged onto the wall of the room and the platform was immersed into the opaque water with white non-toxic painting dye. Mice were trained for 6 days with 4 trials per day and the starting position was in a pseudorandom manner. Probe test was performed 1 day after the last hidden platform training. During probe trials, experimental mice were allowed to swim freely for 1min without platform placing inside the pool but with same distal cues placed in hidden platform trainings. The starting position is at the opposite side of the previous hidden platform location. Escape latency to the platform was calculated as an evaluation of performance of the mice to locate the target. The performance in probe trial was expressed as time spent in the target quadrant with platform location during hidden platform training. Swimming velocity in each trial was also recorded. The value was calculated by EthoVision 3.0.

### Chronic restraint stress model

Mice were housed individually during the whole experimental procedure. For restraint stress groups, mice received a 2-h restraining treatment daily (0800 to 1000 hours) for consecutive 7 days, in 50-ml conical tubes with holes to maintain airflow. After each stress session, mice were returned to their home cages. Mice in control group were handled for around 10 s and returned to the home cage. Body weights were recorded daily before experiment started. At 0800 hours on day 8, the exploratory activity and anxiety-like behavior were monitored in the open field test. On different cohorts of mice, depressive-like behaviors were also studied in the forced swimming and sucrose preference tests.^25^

### Corticosterone measurement

Corticosterone levels in the circulation were measured to determine the activity of the hypothalamic-pituitary-adrenal (HPA) axis in different lines of mice. Mice were divided into two groups, home caged (resting) or stressed. Blood samples from tail tip were collected between 0930 to 1130 hours. For home caged mice, blood was collected rapidly without disturbance before mice removed from their cages. For stressed mice, blood samples were collected immediately after 30-min restraint. Serum was separated by centrifugation and stored at −20°C until assay. Corticosterone levels were measured using the corticosterone ELISA kit (Assay Designs, Ann Arbor, MI, USA) following the manufacturer's instructions.

### Drug treatment

Fluoxetine hydrochloride (Flx, Prozac) was administered to mice at a dose of 10 mg kg^−1^ body weight.^26^ Drugs were prepared freshly in vehicle (0.9% saline) on the day of administration, whereas the control group received the same amount of vehicle only. The animals were orally administered with a feeding needle daily at the same periods of time (0900 to 1100 hours) over the course of 14 days with their body weights recorded every day. On day 15, behavioral assessments were conducted at 0900 to 1100 hours, followed by fluoxetine treatment. Bromodeoxyuridine (BrdU) was administered four times a day after behavioral tests as described above, followed by the last fluoxetine treatment. Animals were killed 24 h after the last BrdU administration.

### BrdU labeling

BrdU labels the dividing cell populations by incorporating into replicating DNA in place of thymidine. For cell proliferation analysis, mice received four injections of BrdU (4 × 75 mg/kg, with 2-h intervals). Animals were killed 24 h after the last BrdU administration.^7^ To determine the survival and differentiation of newborn cells, mice were killed 4 weeks after the last BrdU administration. Brains were collected for BrdU staining and analysis accordingly.

### Sample collection and analysis

#### Sample preparation

For immunohistochemistry, mice were anesthetized with ketamine/xylazine (120 and 10 mg kg^−1^, respectively) and transcardially perfused. Brains were collected, followed by 4% paraformaldehyde fixation overnight at 4 °C, and were stored in 30% sucrose. Serial sections were then cut (40 μm) through each entire hippocampus (Bregma −1.22 to −3.40 mm) using a freezing microtome. Every sixth section was kept for immunohistochemistry.

#### Cell counting

Z-stack confocal images through the entire section were collected using Zeiss 710 confocal microscope to distinguish single cells within clusters. For BrdU and Ki67 staining, all staining-positive cells in the granule cell layer and hilus were counted in each section. The total number of BrdU- or Ki67-labeled cells per section (average of left and right dentate gyrus, DG) was determined and multiplied by 6 to obtain the number of total staining-positive cells per DG.^7^ For doublecortin (DCX), representative images were taken at the middle of suprapyramidal blade of the granule cell layer in each section. DCX-positive cells were categorized according to the morphological grouping criteria described previously.^27^ Density_DCX+cells_=*N*_DCX+cells_/(width_entirely captured GCL_ × thickness_stacked image_). More than 150 DCX-labeled cells were analyzed per animal. For double staining, fluorescence signals were visualized under Zeiss 710 confocal microscope. Colocalization of BrdU with neuronal nuclear antigen (NeuN) or glial fibrillary acidic protein (GFAP) was examined using multiple planes for each BrdU-positive cell on the *z* axis.

### Immunocytochemistry and Western blot analysis

#### Fluorescent staining

After being rinsed with 1 × PBS, sections were pretreated with blocking solution for 30 min at room temperature. Sections were incubated with primary antibodies in diluent at 4°C, for overnight. After washing with 1 × PBS for 10 min 3 times, sections were incubated with secondary antibodies in PBS at room temperature, for 1 h.

#### 3,3'-Diaminobenzidine staining

After being rinsed with 1 × PBS, sections were quenched in 0.3% H_2_O_2_ at room temperature, for 30 min. Sections were blocked and incubated with the primary antibody at 4°C overnight, and, after washing, exposed to biotinylated secondary antibodies at room temperature, for 2 h. Immunoreactivity of samples using biotinylated secondary antibodies was detected with VECTASTAIN Elite ABC kit (Vector Laboratories, Burlingame, CA, USA).

#### Extra steps for BrdU staining

Antigen retrieval was achieved by boiling sections in 10 mM citrate buffer at pH 6.0 for 30 min. Before blocking, sections were exposed to 2N HCl for 30 min for deoxyribonucleic acid hydrolysis, rinsed with PBS and immediately neutralized in boric acid for 15 min.

#### Western blot analysis

Hippocampi were collected and stored in −80°C until further process. Hippocampus was collected and lysed in RIPA buffer (150 mM NaCl, 1.0% NP-40, 0.5% sodium deoxycholate, 0.1% SDS, 50 mM Tris-HCl pH 8.0, EDTA-free protease inhibitors, Roche) for Western blot analysis. Homogenate was centrifuged at 12 000 r.p.m. at 4°C for 15 min. Supernatant was transferred to a new tube and protein concentration was determined by BioRad protein assay. The volumes of the sample used were adjusted to give equal loadings to each lane. Proteins were separated by SDS-PAGE gel and transferred onto a polyvinylidene difluoride membrane. The membrane was blocked for 1h with 5% non-fat dry milk powder in TBST buffer to eliminate non-specific binding sites. The membrane was then incubated with primary antibody in blocking solution for overnight at 4°C Detection was performed with peroxidase-conjugated goat anti-mouse or goat anti-rabbit antibody in 1:2500 dilution. Peroxidase activity was revealed using the enhanced chemiluminescence method. Image J was used for densitometric analysis of blots.

Antibodies used in immunohistochemistry were as follows: BrdU (Ab6326, 1:1000, Abcam, Cambridge, UK), DCX (AB5910, 1:500, Millipore, Billerica, MA, USA), NeuN (MAB377, 1:1000, Millipore), GFAP (Z0334, 1:5000, DAKO, Glostrup, Denmark), pCREB (#9198, 1:1000, Cell Signaling Technology, Danvers, MA, USA) and Ki67 (Ab15580, 1:1000, Abcam, Cambridge, UK). Antibodies used in Western blot analysis included the following: Epac1 and Epac2 (kind gifts from Professor J Bos, 1:2000), PKA (Ab65013, 1:2000, Abcam, Cambridge, UK), phospho-PKA (ABT58, 1:1000, Millipore), BDNF (Sc-546, 1:500, Santa Cruz, Dallas, TX, USA) and α-tubulin (T9026, 1:10000, Sigma-Aldrich, St. Louis, MO, USA).

### Measurement of neurotransmitters using LC-MS/MS

Formic acid (0.5 M) was added to the spinal cord samples at a ratio of 5 ml per gram tissue. Lysates were obtained by homogenization until no debris were observed. The supernatant was collected under twice of centrifugation: 3000 r.p.m. at 4 °C for 15 min and 14 000 r.p.m. at 4 °C for 20 min. Samples were kept at −20 °C before liquid chromatography–electrospray tandem mass spectrometry (LC-MS/MS) measurement was performed as described previously.^28^

### Subject recruitment and assessments

One hundred and forty Chinese AD patients with either NINCDS-ADRDA diagnosis for probable AD, or DSM-IV TR criteria^29^ for AD, were recruited for this study (mean age at study=82.4 years, s.d.=7.6; range=65–97). The patients were recruited through first attendance at the psychiatric clinic of the New Territories East Cluster hospitals and from either Old Age Home or social centers in the community. Four hundred control subjects were recruited from local elderly social residential centers and hostels for the elderly in Hong Kong. Subjects with significant sensory deficits or known neurodegenerative and psychiatric disorders were excluded. All subjects underwent cognitive assessments, including Clinical Dementia Rating (CDR),^30, 31^ Cantonese version of the mini-mental state examination,^32^ Chinese version of the Alzheimer's Disease Assessment Subscale—Cognitive subscale^33, 34^ and delayed recall of list learning test. AD patients were further assessed by the Chinese version of NPI^35^ for the psychiatric symptoms observed in AD. CDR is a standard instrument for the diagnosis and evaluation of the overall severity level of dementia. This rating served as the primary outcome/observation for the current study. In this study, subjects with CDR 0 (not demented), 0.5 (very mild dementia) and 1 (mild dementia) at baseline were recruited. NPI was used to assess the AD patients' neuropsychiatric symptoms in 12 different domains (delusions, hallucination, aggressivity, depression, anxiety, elation, apathy, disinhibition, irritability, aberrant motor behavior, night-time behavior and appetite disturbances). A score for each domain was computed using frequency × severity. During recruitment, the psychiatrists explained the procedure and obtained the informed consent from the subjects and/or their caregivers. The study has been approved by the Clinical Research Ethics Committee of the Chinese University of Hong Kong.

### Genetic analysis of Chinese AD patients

Genomic DNA was extracted from peripheral blood samples using a DNA Extraction Kit according to the manufacturer's instruction (Roche, Nutley, NJ, USA). Two intronic polymorphisms (rs2072115 and rs2074533) in *RAPGEF3* reported to be associated with anxiety and depression were genotyped in this study.^36^ Polymorphisms for *RAPGEF4* in the catalytic domain were selected according to the available genotype information from HapMap, and a set of 10 tagSNPs were selected using R^2^ algorithm among SNPs with minor allele frequencies of at least 5% in Asians.^37^ Genotyping was performed using melting temperature (*T*_m_) shift allele-specific genotyping method.^38^ Briefly, primers were designed to differentiate the WT and mutant base of the SNP by the difference in *T*_m_ between the products of the allele-specific PCR. After DNA amplification, the PCR product was subjected to melting curve analysis using the Roche LightCycler 480 Real-Time PCR System (Roche Applied Science, Penzberg, Germany), and the base of the SNP was distinguished by the melting temperature.

### Statistical analysis

Deviations from the Hardy–Weinberg equilibrium for genotypes at individual loci were assessed by using Pearson *χ*^2^. Statistical analysis of genotype distribution between groups of subjects with different CDR was performed by *χ*^2^-tests. The NPI score was compared among subjects with different genotypes by Student's *t*-test. For all the animal experiments, sample sizes were chosen to ensure adequate power to achieve enough statistical power based on the published articles with the studies and behavioral tests conducted, which are similar to the present study. For the mouse behavioral tests, mice that displayed abnormal behaviors such as seizures immediately before or during the behavioral test would be excluded from the analysis. In animal studies, two-tailed Student's *t*-test or one-way analysis of variance was used for single-factor experiments involving two or more than two groups. For experiments comprising multiple factors, a two-way analysis of variance was used, with *post hoc* analysis (Tukey's multiple comparisons test, unless otherwise stated) between subgroups. A significance level was set to 0.05 for all statistical analyses. Randomization and blinding were not used for animal studies. The data were analyzed using SPSS 17.0 (IBM, Chicago, IL, USA) or Prism 6.01 (GraphPad Software, San Diego, CA, USA). Graphs showing the means and s.e.m. were graphed using Prism.

## Results

### Epac2^−/−^ mice exhibit anxiety- and depressive-like behaviors under normal conditions

**Figure 1:**
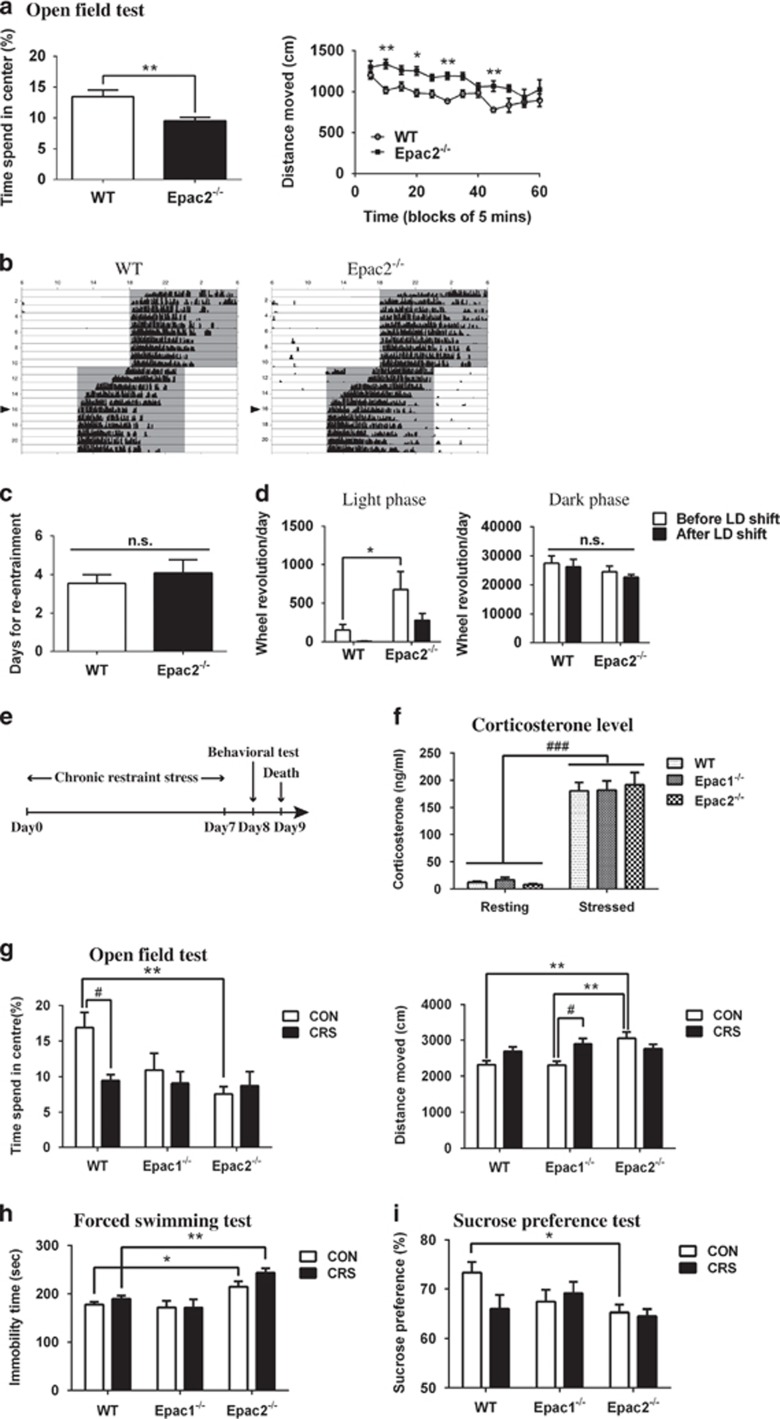
Epac2^−/−^ mice exhibit anxiety- and depressive-like behaviors**.** (**a**) Results of the open field test in wild-type (WT) and Epac2^−/−^ mice. The time spent in the center area and the distance moved during a 1-h period in the open field were measured (for time spent in center, *P<0.05, **P<0.01 by unpaired *t*-test between WT and Epac2^−/−^ mice; for distance moved, genotype effect F_1, 168_=65.87, *P*<0.0001 by two-way analysis of variance (ANOVA), *P<0.05, **P<0.01 by Sidak's multiple comparisons between WT and Epac2^−/−^ mice, *N*=8). (**b**) Wheel-running activities of WT and Epac2^−/−^ mice were showed in representative actograms. The light–dark (LD) cycle was shifted from 0600-1800 to 0000-1200 hours on day 11. Synchronization of the internal circadian clock to the environment LD signal (re-entrainment) happened several days later (arrowheads). (**c**) The average time for re-entrainment was calculated and presented in the bar chart (no significant difference was observed by unpaired *t*-test, *N*=11). (**d**) Wheel-running activity was calculated in light and dark phases, before (days 8–10) and after (days 18–20) the LD cycle shift, respectively (for LD shift effect, F_1, 40_=129, *P*<0.05; for genotype effect, F_1, 40_=9.585, *P*<0.01 by two-way ANOVA. *P<0.05 indicates *post hoc* comparison between genotypes, *N*=11). (**e**) The schematic representation of the chronic restraint stress experimental procedure. Mice of WT, Epac1^−/−^ and Epac2^−/−^ were subjected to either regular handling (CON) or chronic restraint stress (CRS), followed by the open field, forced swimming or sucrose preference test. (**f**) Restraint stress resulted in higher corticosterone levels in mouse serum, with no significant difference among the three genotypes. Blood samples were collected from tail tips of mice in the home cage (resting) or after a 30-min restraint stress (stressed). Corticosterone levels in serum (ng ml^−1^) were measured (for stress effect, F_1, 16_=129.0, ^###^*P*<0.0001; for genotype effect, F_2, 16_=0.02334, *P*=0.9770 by two-way ANOVA, *N*=3-5). (**g**) WT, Epac1^−/−^ and Epac2^−/−^ mice, in CON or CRS groups, were monitored in the open field. The time spent in the center area and the distance moved for the first 10 min were observed (for the time spent in the center, stress effect: F_1, 46_=3.688, *P*=0.0610; genotype effect: F_2, 46_=4.454, *P*<0.05. For the total distance moved, stress effect: F_1, 46_=4.067, *P*<0.05; genotype effect: F_2,46_=4.753, *P*<0.05. *P<0.05 and **P<0.01 indicate *post hoc* comparison between genotypes, ^#^*P*<0.05 and ^**^P<0.01 indicate *post hoc* comparison between control and stressed groups, *N*=8–10). (**h**) In the forced swimming test, the immobility time during the 6 min was plotted (stress effect: F_1,41_=2.358, *P*=0.1323; genotype effect: F_2,41_=15.24, *P*<0.0001. *P<0.05 and **P<0.01 indicate *post hoc* comparison between genotypes, *N*=7–10). (**i**) In the sucrose preference test, mice were given one bottle of water and one bottle of 1% sucrose solution. Their preference to sucrose was expressed as (Δ_weightsucrose_)/(Δ_weight-sucrose_+Δ_weight-water_) × 100 (stress effect: F_1,30_=1.077, *P*=0.3076; genotype effect: F_2,30_=2.357; *P*=0.1120. *P<0.05 indicates *post hoc* comparison between genotypes, *N*=5–8).

To assess the locomotor activity and anxiety-related behavior, WT, Epac1^−/−^, Epac2^−/−^ and Epac1^−/−^;2^−/−^ mice were placed in a novel open field for 1 h. Epac2^−/−^ mice showed a higher locomotor activity, indicated by the longer distance moved, and the less time spent in the center area (Figure 1a). Epac1^−/−^;2^−/−^ mice, but not Epac1^−/−^ mice, showed similar hyperactive and anxiety-like behaviors to those of Epac2^−/−^ mice (data not shown), suggesting that these phenotypes may be attributed to the absence of Epac2. To determine whether the hyperactive phenotype of Epac2^−/−^ mice was triggered by novel environment or constantly exhibited, the locomotor activity on running wheel under different lighting conditions was evaluated (Figure 1b). No significant difference was observed between the WT and Epac2^−/−^ mice in terms of the time for re-entrainment to light–dark cycle shift, which indicated that normal circadian behavior could be maintained in the absence of Epac2 (Figure 1c). Interestingly, Epac2^−/−^ mice showed a significantly higher wheel-running activity in the light phase but not in the dark phase (Figure 1d). The total wheel-running revolution of Epac2^−/−^ mice was similar to that of WT mice (data not shown), but with less precise and increased fragmentations (Figure 1b). This daily activity rhythm of Epac2^−/−^ mice was comparable to those that had been exposed to chronic stress.^39^ To better understand the role of Epac2 in emotional behavior, we employed chronic restraint stress (CRS) model exhibiting hyperlocomotion and anxiety-like behavior^40^ as a positive control (Figure 1e). Restraint stress resulted in higher serum corticosterone levels in all groups; however, there were no significant differences among the three genotypes, whether in resting or stressed condition (Figure 1f). In the open field test, CRS WT mice showed less time spent in the center area and increased locomotor activity. Epac2^−/−^ mice, similar to CRS WT mice, exhibited less time spent in the center area and increased locomotion under control condition, compared with that of WT mice (Figure 1g). In the forced swimming test, Epac2^−/−^ mice displayed aggravated behavioral despairs, as indicated by the increased immobility time compared with those of WT mice under normal condition and the difference was further heightened by CRS (Figure 1 h). In the sucrose preference test, Epac2^−/−^ mice showed reduced preference to sucrose, indicating anhedonia-like behavior, when compared with that of control WT mice (Figure 1i). In all of these behavioral tests, Epac1^−/−^ mice exhibited similar phenotype to those of WT mice. Taken together, Epac2 deficiency induced hyperactivity, anxiety, anhedonia and depression phenotypes in mouse models, indicating a crucial role of Epac2 in emotional controlling.

### Epac2^−/−^ mice with normal spatial learning and memory exhibit deficit in fear conditioning

**Figure 2:**
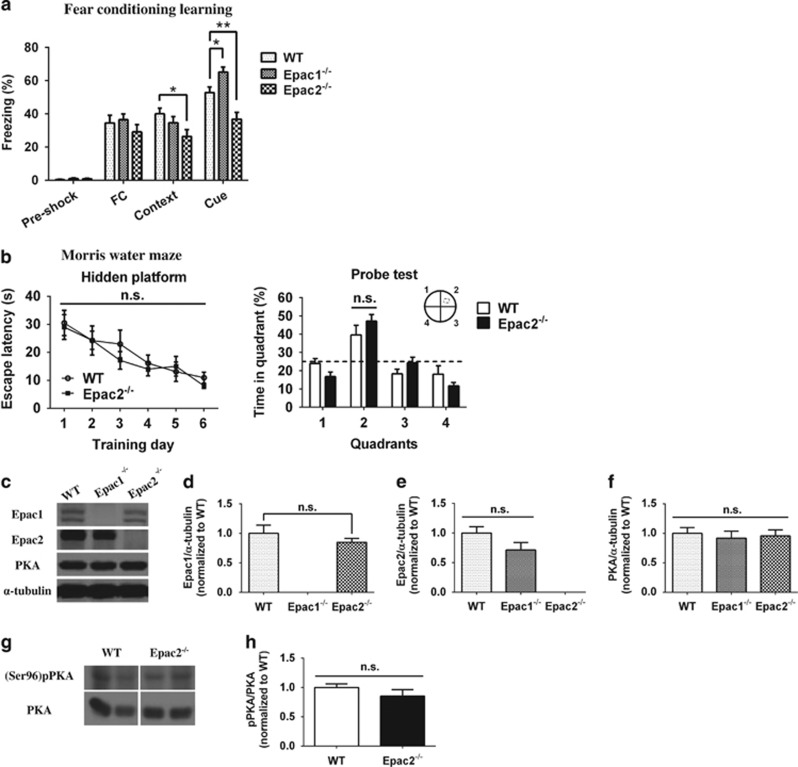
Epac2^−/−^ mice with normal spatial learning and memory exhibit deficit in fear conditioning. (**a**) Epac2^−/−^ mice showed impaired cued and contextual fear memory retrieval. Percentages of the freezing behavior were determined during pre-shock period (free exploration), FC period (all of the 90-s post-shock intervals following the foot shock stimuli) and contextual/cued tests performed on the following 2 days (*P<0.05, **P<0.01 by one-way analysis of variance (ANOVA), *N*=9–11). (**b**) Mice were trained in the hidden platform for 6 days (four trainings per day). Escape latency was calculated as the time from the start to the moment when mice reached the platform. No significant difference was found in escape latency at individual time points in the hidden platform training by unpaired *t*-test. Probe test was conducted 1 day after the hidden platform training. Percentages of the time spent in the 4 quadrants (quadrant 2 as target quadrant in the hidden platform training) were measured. No significant difference was found by unpaired *t*-test. (**c**) Western blots of Epac1, Epac2, protein kinase A (PKA) and the internal control α-tubulin in the hippocampus of wild-type (WT), Epac1^−/−^ and Epac2^−/−^ mice. (**d-f**) Epac1, Epac2 and PKA expression levels in the hippocampus of WT, Epac1^−/−^ and Epac2^−/−^ mice were calculated as the ratios of target proteins to α-tubulin intensities, and data of other genotypes were normalized to that of WT (no significant difference was observed by one-way ANOVA, *N*=6). (**g-h**) Western blots of phosphor-PKA (Ser96) and PKA in the hippocampus of WT and Epac2^−/−^ mice. Ratio of phosphor-PKA to PKA was calculated, and data were normalized to those of WT (no significant difference was observed by unpaired *t*-test, *N*=4–5).

Among the variety of brain structures responsible for controlling emotional response to aversive stress, hippocampus and amygdala are two of the few brain structures that are pivotal in most types of aversive learning.^41^ Two widely used behavioral tasks for evaluating learning and memory were performed on WT and Epac2^−/−^ mice. Pavlovian fear-conditioning test assesses the learning ability to anticipate danger by associating neutral stimuli (context or tone) with aversive events (foot shock). Conditioning to a tone involves projections from the auditory system to amygdala, and conditioning to environment and other contextual cues involves the hippocampus and amygdala.^42^ Compared with the 6 min pre-shock period, foot shock largely increased freezing in animals across all intervals after stimuli, and there were no significant differences among groups of different genotypes (Figure 2a). Interestingly, on the following 2 days, Epac2^−/−^ mice showed significantly reduced freezing response in the contextual test (in the same chamber without tone) and the cued test (in a novel chamber with same tone patterns at the day of training). The deficit in fear conditioning suggested that Epac2 deficiency led to dysfunction of the amygdala and/or hippocampus, which might be correlated to the anxiety and depressive-like phenotype of Epac2^−/−^ mice. On the other hand, in the Morris Water Maze, Epac2^−/−^ mice showed normal spatial learning performance in six consecutive days of the hidden platform training, as well as in the probe test without platform on day 7 (Figure 2b). Western blot analysis revealed that Epac2 was highly expressed in the hippocampus of WT mice and yet absent in that of Epac2^−/−^ mice. Neither compensatory upregulation of Epac1 or PKA nor change of PKA phosphorylation (Ser96) was observed in the Epac2^−/−^ hippocampus (Figures 2c–h).

### Epac2 deletion leads to impaired hippocampal neurogenesis and decreased progenitor cell proliferation

**Figure 3:**
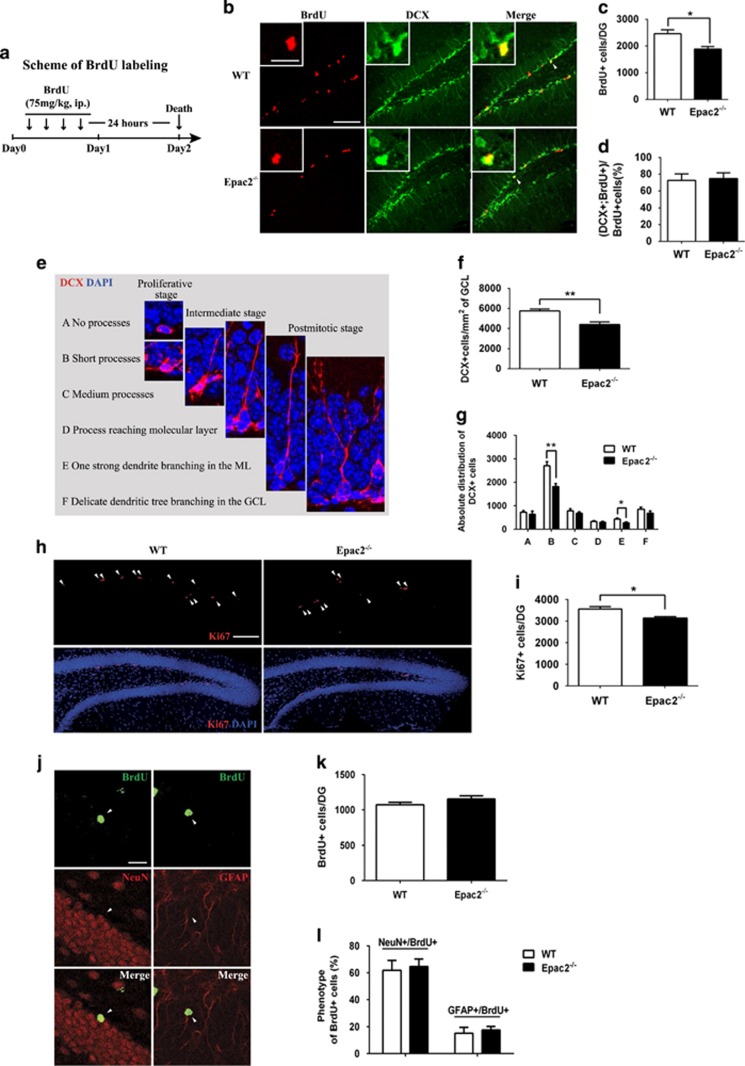
Epac2^−/−^ mice show decreased neural stem/progenitor cell proliferation. (**a**) The schematic representation of the procedure of bromodeoxyuridine (BrdU) labeling. Brain samples were collected 24 h after the last BrdU injection and were co-stained with BrdU and doublecortin (DCX). (**b**) Representative photomicrographs of the adult dentate gyrus (DG) from wild-type (WT) and Epac2^−/−^ mice with BrdU (red) and DCX (green) staining. The majority of the BrdU-positive (BrdU+) cells were located in the subgranular zone of DG. Arrows and insets indicate typical dividing BrdU+ cells with DCX staining (scale bars: 100 μm; inset, 20 μm). Quantitative data were showed as (**c**) the number of BrdU+ cells and (**d**) the percentage of BrdU/DCX double-stained cells in BrdU+ cells in the DG of WT and Epac2^−/−^ mice (*P<0.05 by unpaired *t*-test, *N*=3). (**e**) DCX-positive (DCX+) cells (red) were categorized and counted according to a schema of dendritic morphology (adopted from Plümpe T *et al.*^27^). The six categories, through A to F, represented the development of DCX+ cells in different stages. Quantitative data were showed as (**f**) the total DCX+ cell density and (**g**) the density of DCX+ cells from different categories in the granule cell layer (*P<0.05, **P<0.01 by unpaired *t*-test, *N*=6). (**h**) Representative photographs and (**i**) quantitative data showing Ki67-positive (Ki67+, red) cells in the DG of WT and Epac2^−/−^ mice (scale bar: 100 μm, *P<0.05 by unpaired *t*-test, *N*=4). (**j**) Fate and differentiation of DG BrdU+ cells 28 days after last BrdU injection. Representative confocal microscopic images of cells labeled with BrdU (green) and neuronal nuclear antigen (NeuN, red) or glial fibrillary acidic protein (GFAP, red) in the DG granule cell layer (scale bar: 20 μm). (**k**) Quantitative data show the number of BrdU+ cells 4 weeks after the BrdU labeling. (**l**) Quantitative data show the percentage of NeuN+/BrdU+ or GFAP+/BrdU+ cells 4 weeks after the BrdU labeling. Note that most of BrdU+ cells differentiate into neurons (no significant difference was found by unpaired *t*-test, *N*=4–6).

The role of neurogenesis in pathogenesis of anxiety and depressive disorders has been widely recognized.^5, 43^ Besides, ablation of hippocampal neurogenesis led to impaired contextual fear conditioning but not spatial learning.^4^ These behavioral phenotypes were also observed in the Epac2^−/−^ mice. Therefore, Epac2 may be required for hippocampal neurogenesis that is crucial in regulating behavioral responses under stress condition. To assess this hypothesis, multiple doses of the thymidine analog BrdU were administered to WT and Epac2^−/−^ mice, followed by examination of the cell proliferation in the hippocampus of WT and Epac2^−/−^ mice (Figure 3a). Twenty-four hours after the last BrdU injection, less BrdU-positive (BrdU+) cell clusters were present in the subgranular zone of DG in Epac2^−/−^ mice than those of WT mice (Figures 3b and c). More than 70% of these proliferating cells differentiated further into neuroblasts and immature neurons expressing the marker of DCX (Figure 3d). Meanwhile, we observed a presence of empty regions where no DCX+ cells were found in Epac2^−/−^ DG. To better understand the observed condition, a morphological categorization method was adopted^27^ (Figure 3e), so that the density of total DCX+ could be quantified and the cell phases (proliferative, intermediate or postmitotic) be distinguished at the same time. Within the identical area, the number of DCX+ cells was significantly decreased in Epac2^−/−^ mice compared with that of WT mice (Figure 3f). Although cell numbers in each category were reduced, the most obvious difference came from type B (DCX+ cells with short and plump process) and type E (DCX+ cells with one strong dendrite branching in the molecular layer; Figure 3g). These data indicated that there was impairment in the neuronal progenitor cells at proliferative and postmitotic stages in the absence of Epac2. The expression of endogenous proliferative marker Ki67 was also significantly reduced in the DG of Epac2^−/−^ mice (Figures 3h and i). Taken together, the results of BrdU labeling, DCX and Ki67 staining results consistently suggested the impairments of hippocampal progenitor cell proliferation and neurogenesis in Epac2^−/−^ mice. Survival and differentiation of the BrdU+ cells were examined 28 days after the last injection of BrdU (Figures 3j–l). Note that the number of BrdU+ cells was largely decreased when compared with that at 24 h post injection, whereas no significant difference was observed between WT and Epac2^−/−^ mice (Figure 3k). The cell fate of BrdU+ cells was also determined by double staining with BrdU and neuronal marker NeuN or astrocytic marker glial fibrillary acidic protein (Figure 3j). More than 60% of the BrdU+ cells differentiated into neurons as indicated by BrdU and NeuN co-staining. No significant difference in the percentage of BrdU+ neurons or astrocytes was found between WT and Epac2^−/−^ mice (Figure 3l). These data demonstrated that Epac2 deficiency did not affect the survival and differentiation of adult-born cells in DG *in vivo*.

### SSRI treatment increases hippocampal cell proliferation and ameliorates anxiety-like behavior in Epac2^−/−^ mice

**Figure 4:**
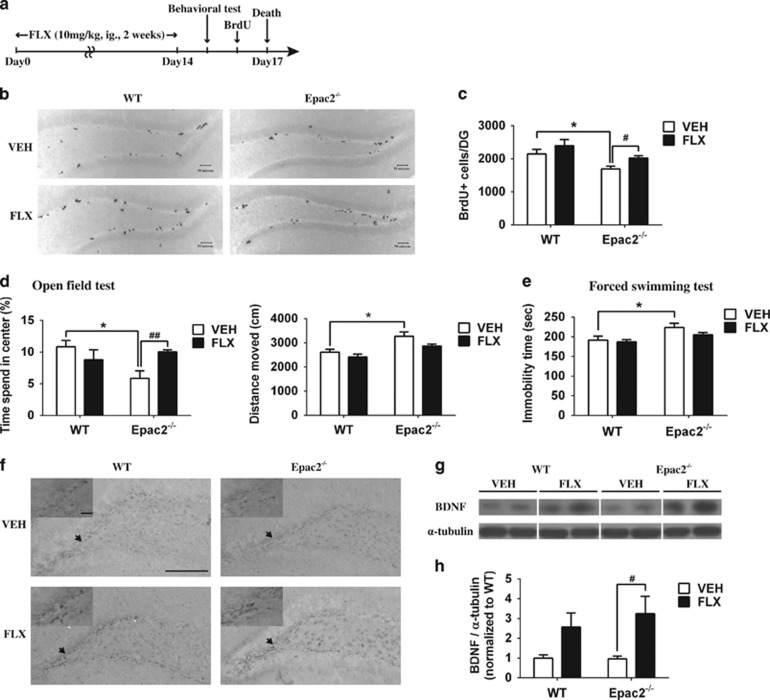
Selective serotonin reuptake inhibitor (SSRI) treatment with neurogenic effect ameliorated anxiety-like behavior in Epac2^−/−^ mice. (**a**) Timeline of drug treatment, behavioral tests and bromodeoxyuridine (BrdU) labeling. (**b**) Representative photomicrographs and (**c**) quantitative data show the BrdU+ cells in the dentate gyrus (DG) of wild-type (WT) or Epac2^−/−^ mice with vehicle or fluoxetine treatment (treatment effect: F_1,16_=5.078, *P*<0.05; genotype effect, F_1, 16_=10.57, *P*<0.01; *P<0.05 indicates *post hoc* comparison between genotypes, ^#^*P*<0.05 indicates *post hoc* comparison between vehicle and fluoxetine-treated groups; *N*=3–5, scale bar: 50 μm). (**d**) Open field test and (**e**) forced swimming test were conducted after a 2-week saline (VEH) or fluoxetine (FLX) treatment (for time spent in the center, treatment effect: F_1,16_=0.8835, *P*=0.3612; genotype effect: F_1,16_=2.780, *P*=0.1149. For the distance moved, treatment effect: F_1,16_=5.563, *P*<0.05; genotype effect: F_1,16_=18.98, *P*<0.001. *N*=5 in the open field test. For the forced swimming test, treatment effect: F_1,34_=2.057, *P*=0.1607; for genotype effect, F_1,34_=9.183, *P*<0.01; *N*=9-10. *P<0.05 indicates *post hoc* comparison between genotypes, ^##^*P*<0.01 indicates *post hoc* comparison between vehicle and fluoxetine-treated groups). (**f**) Representative photomicrographs of pCREB staining in the DG from vehicle or fluoxetine-treated WT or Epac2^−/−^ mice (*N*=3, scale bars: 100 μm; inset, 20 μm). (**g**) Brain-derived neurotrophic factor (BDNF) expression levels in the hippocampus of vehicle or fluoxetine-treated mice were detected by Western blot analysis. (**h**) α-tubulin was used as the internal control. Data from other groups were normalized to vehicle-treated WT group (treatment effect, F_1,16_=11.18, *P*<0.01; genotype effect, F_1,16_=0.3050, *P*=0.5884; ^#^*P*<0.05 indicates *post hoc* comparison between vehicle and fluoxetine-treated groups; *N*=5–6).

Antidepressant fluoxetine, a selective serotonin reuptake inhibitor, promotes hippocampal neurogenesis in adult rodents,^7^ and it mainly targets early amplifying progenitor cells.^26^ As a defect in amplifying progenitor cells was observed in Epac2^−/−^ mice with anxiety and depression, we hypothesized that these behavioral phenotypes of Epac2^−/−^ mice could be rescued by neurogenic fluoxetine treatment. WT and Epac2^−/−^ mice were chronically administered with fluoxetine for 2 weeks (Figure 4a). To examine the drug effect on neurogenesis, BrdU was administered 24 h before killing the mice. Representative photomicrographs and quantitative data showed that in the vehicle-treated group Epac2^−/−^ mice had less BrdU+ cells than WT mice. BrdU+ cells in the subgranular zone of WT mice were slightly increased after fluoxetine treatment, whereas BrdU+ cells in fluoxetine-treated Epac2^−/−^ mice were significantly reversed (Figures 4b and c). Fluoxetine significantly ameliorated anxiety-like behavior of Epac2^−/−^ mice, indicated by increased time spent in the center area of the open field, although no positive effect was observed in the fluoxetine-treated WT mice. Meanwhile, the hyperactive phenotype of Epac2^−/−^ mice was moderately reversed by fluoxetine treatments (Figure 4d). Similarly, vehicle-treated Epac2^−/−^ mice showed significantly higher immobility than their WT counterparts, and fluoxetine only showed marginal effects in either WT or Epac2^−/−^ mice (Figure 4e). The cAMP-CREB cascade profoundly contributes to adult neurogenesis and to SSRI-mediated neurogenesis.^44^ In agreement with the neurogenic effect, administration of SSRI significantly increased the expression of pCREB (Ser133) in the hippocampal lysates of WT and Epac2^−/−^ mice (Figure 4f). Likewise, BDNF proteins were significantly elevated in fluoxetine-treated Epac2^−/−^ mice, and a trend of increase was also observed in WT mice (*P*=0.0645, Figures 4g and h).

### Epac2 deficiency leads to reduced GABA concentrations in the hippocampus

To investigate the potential role of Epac2 in regulating major neurotransmitters, concentrations of GABA, dopamine, norepinepherine, glutamate, serotonin and serotonin metabolite (5-hydroxyindoleacetic acid, 5-HIAA) were determined by LC-MS/MS in the hippocampal homogenates simultaneously (Table 1, *N*=5-6).^28^ The serotonin (5-HT) turnover rate was calculated as the ratio of 5-HIAA/5-HT. Epac2^−/−^ mice showed the normal level of 5-HT and 5-HIAA, and maintained the normal 5-HT turnover rate in the tissue homogenates of hippocampus, indicating that the synthesis and elimination of 5-HT might not be affected by deleting the *Epac2* gene. Similarly, dopamine, norepinephrine and glutamate concentrations in the Epac2^−/−^ hippocampus were comparable to those of WT. Interestingly, the GABA concentration in hippocampal homogenates from Epac2^−/−^ mice (1157.52 ng ml^−1^) was significantly lower (~15%) than that in the WT mice (1334.36 ng ml^−1^), suggesting a critical role of Epac2 in modulating GABAergic neurons in the hippocampus. Besides, we measured GABA levels in the hippocampus of Epac2^−/−^ mice treated with vehicle or fluoxetine. Surprisingly, the reduced GABA level in Epac2^−/−^ mice did not get corrected by chronic fluoxetine treatment (Supplementary Figure 1).

### Non-coding *RAPGEF4* SNPs are associated with cognitive decline and mood disturbance in AD patients

Previously reported SNPs in *RAPGEF3* gene^36^ were screened and no significant association between these SNPs and the risk of AD or NPI score was observed (data not shown). Interestingly, significant differences in genotype distribution for rs3820841 (intron 17) and rs3769219 (intron 23) in *RAPGEF4* were observed among normal control subjects (CDR 0), subjects with very mild dementia (CDR 0.5) and AD patients (CDR 1; Table 2). There was no difference in genotype distribution of *RAPGEF4* rs17746510 (intron 22) between normal subjects and AD patients; however, significant difference was observed between subjects with CDR 0.5 and CDR 1. The NPI score in different domains was compared among AD patients with different genotypes (Table 3). AD patients with GG genotype of rs17746510 was associated with a higher NPI score in apathy and mood disturbance, but not in domains in behavior, psychosis and sleep, suggesting that they encountered more complains in emotion-related domains.

## Discussion

There is a close inter-relationship between mood and cognition, both of which have equally disabling effects on patients. Understanding the common molecular and cellular mechanisms underlying the progression of cognitive decline and concomitant affective traits, such as anxiety, apathy and depression, is critical in developing therapies for patients with mood disorders or neurodegenerative diseases such as AD. In the present study, we showed that Epac2 deletion, but not Epac1 deletion, led to emotional disturbance, indicated by anxiety, hyperactivity, anhedonia and depressive-like behaviors in mouse models. Epac2^−/−^ mice exhibited normal spatial learning but reduced fear conditioning. On the basis of the observation that SSRI treatment upregulated hippocampal neurogenesis and rescued the behavioral deficits of Epac2^−/−^ mice, we conclude that these abnormalities in emotion and cognition of Epac2^−/−^ mice were associated with impaired neurogenesis, especially neural progenitor cell proliferation in the DG. Furthermore, the abnormal emotional behavior and neurogenesis impairment were associated with a mild deficit of GABA content in the hippocampus of Epac2^−/−^ mice. Finally, we showed association of several polymorphisms in *RAPGEF4* with cognitive decline and mood disturbance in AD patients.

### Epac2 deficiency induced emotional disturbance and cognitive impairment in mouse model

Altered expression and activation of Rap1 and Epac2 were shown in a post-mortem study on depressed suicide victims,^15^ but the underlying mechanism is poorly understood because of the lack of clinical samples. In the present study, *in vivo* animal study was performed by using Epac knockout mice. Epac2^−/−^ mice were highly stress-reactive, mimicking patients with depression and comorbid anxiety. In the open field test, Epac2^−/−^ mice showed anxiety-like phenotype, similar to that of WT mice exposed to chronic mild stress. This phenotype was also observed in Epac1^−/−^;2^−/−^ mice, but not in Epac1^−/−^ mice (data not shown). To further confirm our observation, diurnal wheel-running activity of mice in their home cages was recorded, as stressed mice have been shown to display a higher percentage of wheel-running activity in the light phase as well as fragmented wheel-running pattern.^39^ Similar to stressed mice, Epac2^−/−^ mice displayed a significantly higher activity during the light (inactive) phase, although no such difference was observed during the dark (active) phase compared with WT mice. These data suggested that Epac2 might have a role in anti-anxiogenic effects under mild stressor, such as a novel environment and altered lighting. Our finding was not in agreement with other studies in which Epac2^−/−^ mice did not show anxiety-like behavior in the open field test.^45, 46^ These behavioral discrepancies may because of the intrinsic factor, like mouse strain, and the environmental factors such as lighting conditions.^47^ On the other hand, disturbance to the circadian timekeeping system is closely linked with mood disorders and cognitive dysfunction.^48^ Recently, cAMP/Epac signaling was reported to sustain the circadian rhythm pacemarker in the suprachiasmatic nuclei, potentially by activating CREB and *Per* transcription.^49^ In the present study, the abnormal circadian rhythm or re-entrainment after light–dark cycle shift in Epac2^−/−^ mice was undetected, indicating that Epac1 and Epac2 may have redundant roles or functional compensation in physiological circadian clockwork (Supplementary Figure 2).

Furthermore, Epac2^−/−^ mice, but not Epac1^−/−^ mice, showed despair and apathy behaviors in the forced swimming and sucrose preference tests, supporting a vital role of Epac2 in emotional appraisal. Abnormal stress hormone corticosterone levels may be a potential explanation of anxiety,^50^ anhedonia and behavioral despair.^51^ Epac has been reported to couple adrenocorticotropic hormone-mediated cortisol secretion from the adrenal zona fasciculate.^52^ In the present study, no significant difference in serum corticosterone levels was observed among the three genotypes, either in the resting or stressed condition. Besides, the weight of adrenal glands of Epac2^−/−^ mice was similar to that of WT (data not shown). These results suggested that the anxiety- and depressive-like phenotypes induced by Epac2 deletion might be independent of the basal corticosterone maintenance and its secretion under stress. However, we do not exclude the potential involvement of Epac2 in the negative feedback regulation of the HPA axis regarding its role in hippocampal neurogenesis.^5^

Cognition interplays with stress pathogenesis because of the common underpinning cerebral regions, such as hippocampus and amygdala.^41^ Previous studies have showed that the Epac agonist, 8-pCPT-2'-O-Me-cAMP, improves hippocampus-dependent conditioned memory retrieval.^17, 18^ However, such Epac agonist activates both Epac1 and Epac2.^53^ Here we observed that Epac2, but not Epac1, was required for contextual and cued fear-conditioning memory retrieval, indicating that the effect of Epac agonist previously found in hippocampus-dependent fear memory retrieval was because of the activation of Epac2. Besides, Epac2^−/−^ mice showed reduced freezing behavior in cued and contextual tests 7 days after training, compared with WT mice, indicating that Epac2 not only has a role in intermediate-term memory retrieval but also potentially has a lasting effect on consolidation (Supplementary Figure 3). Such observation partially contradicts previous report that knockdown of Epac2 by intrahippocampal short interfering RNA transfection did not alter freezing behavior 17 days after the training.^54^ We speculated that this discrepancy might have been resulted from either the degree (knockdown versus knockout) of loss of Epac2 function, or other differential experimental schemes. The normal performance in the Morris Water Maze test indicated that such memory deficits of Epac2^−/−^ mice are specifically related to emotion and fear. Accordingly, long-term potentiation was triggered and recorded in CA3–CA1 of cultured hippocampal slices and no significant difference was observed between WT and Epac2^−/−^ brain slices (data not shown). One of the interpretations of these two results is that Epac2 may influence learning and memory primarily in tasks that involve significant emotional arousal or coactivation of the hippocampus and the amygdala, which is supported by the relatively high expression level of Epac2 in the hippocampus and amygdala in humans.^14^ Another interpretation based on the difference in nature of these two learning tests is that contextual conditioning learning is induced during exposure to a novel environment, whereas spatial learning in the Water Maze Test occurs in the same environment repetitively. Novelty exploration leads to the enhanced long-term potentiation in the DG,^55, 56^ which is suggested to involve distinct long-term potentiation in the medial perforant path/DG connection mediated by new neurons.^4^ In fact, a lower rate of neurogenesis in the DG has long been shown to correlate with hyperlocomotor activity in a novel environment.^57^ Such hypothesis is in line with our other findings that Epac2^−/−^ mice exhibited hyperactivity and anxiety in the open field test, in which the animals were introduced to a novel environment, and Epac2^−/−^ mice had impaired hippocampal neurogenesis.

### Epac2 deficiency induced suppression on hippocampal neurogenesis

Epac2 deletion negatively regulated hippocampal neural progenitor proliferation *in vivo*, which had a pivotal role in behavioral responses to novelty, emotional controlling and cognitive functions under the novel and stressful condition.^4, 5, 58^ To assess whether the abnormal behaviors of Epac2^−/−^ mice might be related to impaired neurogenesis, the antidepressant with neurogenic effects, fluoxetine, was administered to examine whether this could rescue the behavioral defect in Epac2^−/−^ mice. As expected, fluoxetine treatment ameliorated retarded hippocampal neurogenesis, indicated by the increased BrdU labeling in DG. Fluoxetine treatment on Epac2^−/−^ mice significantly reversed anxiety-like behavior indicated by the increased time spent in the center area of an open field. After a short period of fluoxetine treatment, the Epac2^−/−^ group exhibited a trend of decreased hyperactivity in the open field test and immobility in the forced swimming test; therefore, it is postulated that a prolonged treatment or higher dosages of fluoxetine would have shown a significant effect.^59^ The data above indicated that the anxiety and depressive behaviors of Epac2^−/−^ mice were associated with the impaired neurogenesis. Besides, no significant change of behaviors in WT mice following the identical antidepressant treatment was observed. We speculated that fluoxetine treatment reduced anxiety on stressed but not on normal animals,^60^ similar to the report that antidepressants have profound mood-altering effects in patients rather than in healthy individuals.^61^ The mechanisms of fluoxetine action involve multiple molecular pathways, of which the most well studied is through activation of serotonergic receptors,^62^ cAMP-CREB signaling pathway^44^ and increased production of BDNF.^63^ In agreement with the above studies, we showed that the anxiolytic and neurogenesis effect of fluoxetine on Epac2^−/−^ hippocampus was associated with upregulation of pCREB (Ser^133^) and BDNF expression. Although Rit, an effector of Epac proteins, can lead to activation of the CREB by phosphorylation,^64^ and the Epac agonist can trigger CREB phosphorylation in suprachiasmatic-nucleus brain slices,^49^ we did not find differences in baseline pCREB or BDNF levels between WT and Epac2^−/−^ hippocampus, indicating the cAMP/CREB/BDNF pathway in hippocampus remained intact in the absence of Epac2.

There are several potential explanations for the mechanistic role of Epac2 in regulating hippocampal neurogenesis. First, the Rap1/B-Raf/ERK signaling pathway downstream to various growth factor receptor tyrosine kinases controls cell proliferation, differentiation, migration and survival.^65, 66^ Mice with B-Raf mutation show reduced proliferation in the developing cortex of E14.5.^66^ Although the perinuclearly localized endogenous Epac1 could not mediate Rap1-induced ERK activation, translocation of Epac1 to the plasma membrane by adding a membrane-targeting motif allows it to activate the Rap1/ERK pathway.^67^ On the other hand, the presence of N-terminal cAMP-binding domain in Epac2 enables it to localize to the plasma membrane,^68^ which suggests a potential role of Epac2 in regulating the Rap1/B-Raf/ERK pathway.

Furthermore, although a lower number of neural progenitor cells was initially produced in the adult hippocampus of Epac2^−/−^ mice, eventually no difference between genotypes was shown with regard to the number of these cells that were able to survive till 28 days after BrdU labeling. Similar to our observation, the discrepancy between proliferation and neuronal cell survival, indicated by short-term and long-term BrdU labeling, was also reported by other groups.^57, 69^ There are two potential explanations to this phenomenon. First, a large portion of excess neurons is eliminated through apoptosis during neurogenesis.^70, 71^ Activation of Epac induced apoptosis in cultured primary mouse cortical neurons via upregulation of pro-apoptotic Bcl-2 interacting member protein (Bim),^72^ suggesting that Epac2 deficiency might lead to a decrease in the newborn neuron-programmed cell death, which resulted in impaired progenitor cell proliferation with the similar levels of cell survival. Second, there may be a compensatory response, during the development of Epac2^−/−^ mice, that results in less cell death or less competition for trophic support to maintain the appropriate number of new neurons compared with WT mice. Nevertheless, these speculations do not weaken our finding that deficits in cell proliferation and early-stage hippocampal neurogenesis are associated with the depressive-like behavior and fear-related cognitive deficit of Epac2^−/−^ mice. The newborn neurons within 1–3 weeks after their birth were shown to have a unique role in hippocampal-dependent memory formation.^73^ These cells have high excitablility and reduced GABAergic inhibition;^74, 75, 76^ therefore, they would be preferentially activated by specific activity patterns entering the DG. The detailed morphological characterization of DCX+ cell in WT and Epac2^−/−^ DG indicated that the Epac2 deficiency might mainly affect the newborn cells within this period,^77^ which are associated with the behavioral phenotypes in Epac2^−/−^ mice.

In addition, adult neurogenesis is tightly regulated through the interaction between neural stem/progenitor cells and their niche, in which neurotransmitters are crucial environmental signals. Here we measured the most common neurotransmitters associated with mood disorders in the hippocampus of WT and Epac2^−/−^ mice. Although it is widely accepted that abnormality in the serotonergic system is involved in the onset and the course of depression, as well as impaired hippocampal neurogenesis,^78^ we observed the normal level of 5-HT and the 5-HT turnover rate in the hippocampus of Epac2^−/−^ mice. Interestingly, GABA concentrations were significantly reduced in the homogenate of Epac2^−/−^ hippocampus. Patients with major depressive disorders have reductions of GABA in the occipital^79^ and the prefrontal cortex,^80^ assessed using proton magnetic resonance spectroscopy. Although there is a lack of GABA measurement in the hippocampus of depressed individuals, preclinical studies showed that chronic stress reduced the number and rhythmic firing of parvalbumin-positive interneurons in the hippocampus,^81^ indicating that function of GABAergic neurons is tightly associated with stress-related hippocampal activity. In rodent studies, chronic stress functionally impaired the hippocampal GABAergic neurotransmission, which was partially reversed by SSRI escitalopram.^82^ Surprisingly, SSRI fluoxetine treatment did not correct the reduced GABA level in Epac2^−/−^ mice, although it was able to reduce the anxiety-like behavior and improve the deficit in hippocampal cell proliferation in these mice. On the basis of this observation, we speculated that the antidepressive and pro-neurogenic effects of fluoxetine treatment in Epac2^−/−^ mice are, at least partially, acting through the serotonin system. On the other hand, it is recently discovered that GABA_B_ receptor deletion or inhibition increased cell proliferation in the DG.^83^ Epac activation reversed the GABA_B_ activation-induced vesicle recruitment suppression in the presynaptic terminal of glutamatergic neurons.^84^ Therefore, Epac, especially Epac2, has a potential role in balancing against the GABA_B_ receptor inhibition in transition of neural stem/progenitor cells from quiescent to proliferative status. It remains unclear whether the reduction of hippocampal GABA levels is the causal factor of depression and neurogenic deficits of Epac2^−/−^ mice, or only a compensatory effect to rescue the imbalance of GABAergic downstream signaling.

### Polymorphisms in the *RAPGEF4* gene were associated with cognitive decline and mood disturbance in patients

AD patients are observed to exhibit comorbid neuropsychiatric symptoms, such as apathy, aggressivity, depression, anxiety, irritability, dysphoria, aberrant motor behaviors, delusions, hallucinations, disinhibition, sleep-pattern abnormalities and appetite disturbance.^85^ On the other hand, stress steroids impair cognitive function, and a history of depression increases the risk of later development of AD.^86, 87^ The high comorbidity implies that progression of cognitive impairment and mood disturbance in AD share common cellular and molecular cascades, for example, neurogenesis.^88^

We initially demonstrated the significant association of three intronic *RAPGEF4* SNPs near the catalytic domain coding sequences with the risk of AD in Chinese patients, suggesting its role in the pathogenesis of AD and development of dementia. Reduced Epac2 mRNA was shown in the frontal cortex and hippocampus regions of Alzheimer's brains.^89^ Considering the crucial role of Epac2 in synapse remodeling and dendrite morphology,^90^ decreases in Epac2 expression in the post-mortem brain of AD patients may result from loss of neurons and synapses in the late stage of AD. Besides, it is speculated that Epac proteins may also involve in the AD progression because Epac mediates secretion of the non-amyloidogenic protective form of APP, sAPPα, induced by 5-HT_4_ receptor activation *in vitro.*^91, 92^

Furthermore, we found a significant association between several SNPs in *RAPGEF4* and NPI scores for apathy and mood disturbance in AD patients. Subjects with the GG genotype of *RAPGEF4* SNP rs17746510 were more susceptible to the presentation of disturbance in mood and apathy, but not sleep disturbance or other psychotic symptoms. Proteins of Epac2 but not Epac1 were increased in the prefrontal cortex and the hippocampus of depressed suicide victims, together with the attenuated activation and expression of Rap1.^15^ This is in line with our observation in the mouse model that Epac2 has a predominant role in emotion controlling. Owing to the limitation of sample availability, we did not show functional attributes of these SNPs in the human central nervous system. However, *in silico* analyses by the use of Regulome database suggested that *RAPGEF4* SNP rs17746510 falls in highly conserved consensus-binding sequences (TGACAG) of transcription factors such as MEIS1 (myeloid ecotropic viral integration site 1).^93^ Interaction between MEIS1 and Evolutionary Conserved Regions 1 enhancer regulates the tissue-specific expression of neuropeptides such as substance P in the central amygdala,^94^ which has a role in the modulation of fear and anxiety. We speculated that the *RAPGEF4* SNP rs17746510 might greatly influence the expression pattern of Epac2 in the central nervous system.

Taken together, we provided evidence that mutations of the *RAPGEF4* gene have cross-species conservative function on cognitive and emotional regulation. In conclusion, we showed that (1) Epac2 had a dominant role in emotional cognitive function and mood control over Epac1; (2) impaired hippocampal neurogenesis was among the most important factors that related to anxiety, depressive-like behaviors and fear-related cognitive deficits in Epac2-deficient mice; (3) the neurogenesis deficit might be attributed to the indispensable role of Epac2 downstream to GABAergic regulation of recruiting quiescent cells to the active proliferative cell pool. Investigation of the mechanistic role of Epac2 in the cause and consequence nature of the neurogenesis–synaptic function is being carried out. Whether there is a primary and secondary cause underlying the mood disturbance and memory deficits in the absence of Epac2 remains a challenge for future research. As abnormal adult neurogenesis is characterized in a number of cognition- and emotion-related psychiatric disorders, understanding the function of Epac sheds light on novel treatments for neurodevelopmental and neuropsychiatric diseases.

## Figures and Tables

**Table 1:** LC-MS/MS measurement of neurotransmitters in the hippocampus of WT and Epac2^−/−^ mice

	*WT*	*Epac2*^−/−^	P*-value*
5-HT (ng ml^−1^)	1.31772	1.32372	0.8792
5-HIAA (ng ml^−1^)	22.6468	22.8780	0.3113
5-HT turnover	17.4129	17.3516	0.9270
Norepinephrine (ng ml^−1^)	189.196	176.364	0.2178
Dopamine (ng ml^−1^)	17.8403	17.9343	0.4695
Glutamate (ng ml^−1^)	7895.45	7557.04	0.3144
GABA (ng ml^−1^)	1334.36	1157.52	*0.0258

Abbreviations: 5-HIAA, 5-hydroxyindoleacetic acid; 5-HT, serotonin; LC-MS/MS, liquid chromatography–electrospray tandem mass spectrometry; WT, wild type. *P<0.05 by unpaired *t*-test between WT and Epac2^−/−^ mice.

**Table 2:** Significant association between RAPGEF4 SNPs and cognitive decline

*RAPGEF4 SNP*	*Subjects*	*Genotype*			P*-value*
*rs3769219*		*TT*	*TC*	*CC*	
	CDR 1	0 (0%)	12 (9.4%)	115 (90.6%)	**0.003
	CDR 0.5	0 (0%)	25 (22.5%)	86 (77.5%)	
	CDR 0	0 (0%)	45 (24.5%)	139 (75.5%)	
					
*rs3820841*		*TT*	*TC*	*CC*	
	CDR 1	35 (25.4%)	62 (44.9%)	41 (29.7%)	**0.001
	CDR 0.5	37 (22.8%)	49 (30.2%)	76 (46.9%)	
	CDR 0	42 (17.7%)	71 (30.0%)	124 (52.3%)	
					
*rs17746510*		*TT*	*TG*	*GG*	
	CDR 1	48 (34.8%)	72 (52.2%)	18 (13.0%)	0.167
	CDR 0	93 (39.2%)	101 (42.6%)	43 (18.1%)	
	CDR 1	48 (34.8%)	72 (52.2%)	18 (13.0%)	*0.036
	CDR 0.5	62 (38.5%)	63 (39.1%)	36 (22.4%)	
	Combined				
	CDR 1	48 (34.8%)	72 (52.2%)	18 (13.0%)	0.13
	CDR 0.5	62 (38.5%)	63 (39.1%)	36 (22.4%)	
	CDR 0	93 (39.2%)	101 (42.6%)	43 (18.1%)	

Abbreviations: CDR, Clinical Dementia Rating; SNP, single-nucleotide polymorphism. *P<0.05, **P<0.01 by *χ*^2^-tests.

**Table 3:** The association between genotypes of RAPGEF4 SNP rs17746510 and neuropsychiatric symptoms (presented by NPI score) in AD patients

*NPI score domain*	*NPI score*^a^		
	*TT/TG*	*GG*	P*-value*
Behavior	0.316	0.889	0.219
Psychosis	0.065	0	0.568
Mood	1.88	4.778	*0.03
Apathy	1.213	3	*0.049
Sleep	0.382	0.889	0.279

Abbreviations: AD, Alzheimer's disease; NPI, Neuropsychiatric Inventory; SNP, single-nucleotide polymorphism. *P<0.05 by student's *t*-test.

a NPI score is calculated by frequency of symptom occurrence and severity.
