# Supplementary material for: Anxiety and depression with neurogenesis defects in exchange protein directly activated by cAMP 2-deficient mice are ameliorated by a selective serotonin reuptake inhibitor, Prozac
Source: Transl Psychiatry. 2016 Sep 6;6(9):e881–. doi: 10.1038/tp.2016.129 (PMC5048194; doi:10.1038/tp.2016.129)
Supplement: Supplementary Materials and Methods [file tp2016129x1.pdf]

Supplementary Materials and Methods

Open field test

Open field test is a well-established behavioral assessment to the locomotor activity and anxiety related behavior. The open field was a transparent (26 cm length × 26 cm width × 40 cm height) box with equivalent mouse bedding [1]. The 10 cm × 10 cm square in the center of the box was defined as center arena. Four mice were detected in individual boxes at the same time and cardboards were placed in between to avoid disturbance. Mice were allowed to move freely in the open field for 10 minutes or 1 hour. Their movements were video tracked and analyzed by EthoVision 3.0 (Noldus Information Technology bv). Total distance moved and time spent in center area were calculated accordingly.

Forced swimming test

A transparent cylinder (25 cm height × 10 cm diameter) was filled up with water up to a height of 10 cm, as described earlier [2]. Fresh water was equivalent to the room temperature and changed for individual mouse. The camera was oriented for horizontal viewing. A sheet of cardboard was placed in-between the two beakers so that the mice could not see each other. The movement of mice was recorded by software EthoVision 3.0 and divided into 3 kinds: immobility, mobility and strong mobility. Duration of immobility time was recorded in total 6 minutes.

Sucrose preference test

Mice were individually housed and given two bottles of liquid: a bottle with plain water and the other one with 1% sucrose. Bottles were reversed every 12 hours.

After 3 days of habituation, all bottles were removed at 10:00 A.M.. After 12 hours water deprivation, mice were given access to bottles of water and sucrose, in the reversed location. The bottles were reversed again 1 hour later and weighed on the following morning. Sucrose preference was expressed as (Δweightsucrose)/(Δweightsucrose + Δweightwater) × 100 [3].

Circadian rhythm assessment

This experiment was performed in Hong Kong University of Science and Technology, with 12:12 light/dark cycle (L 6:00 A.M. to 6:00 P.M.). Mice were housed individually in cages with running wheels linked to detectors [4]. Day 1-10 was the habituation period. On day 11, the dark cycle was advanced by 6 hours (L 12:00 A.M. to 12:00 P.M.) and mice were allowed to re-entrain for another 10 days. Time spent for adapting to the new light dark cycle was recorded. Average wheel running activity in light phase and dark phase was calculated before light dark shift (day 8 - day10), and after light dark shift (day 18 - day 20). Water and food were provided *ad libitum*. The wheel running activity was recorded and analyzed using the Clocklab software (Actimetrics, Wilmette, IL, USA).

Pavlovian fear conditioning

This experiment was performed as previously described [5]. Briefly, on day 1, mouse received a training session of 13.5 minutes in conditioning chamber (25 cm × 25 cm × 25 cm), where the mouse could freely explore in the chamber. The training consisted of a habituation period of 6 minutes, followed by 3 times of paired presentations of tone (conditional stimuli) and foot shock (unconditional stimuli). The foot shock (0.5mA) was applied to the floor grid of the chamber during the final 2 seconds of the

tone (30 sec, 4 Hz, 80 dB). There was 2 minutes rest every time after the tone-shock pairing. The chambers were wiped and cleaned with 70% ethanol between training sessions. On day 2, fear conditioning to the context was assessed by returning the mice to the same chamber for 8 minutes, without tone or shock stimuli. On day 3, fear to the tone conditional stimuli was assessed in novel chamber with explicit cue as day 1, in the absence of foot shock. The chambers were cleaned with diluted creamy soap to remove olfactory debris. The contextual and cued tests scheme was applied again to the mice 1 week after the training. EthoVision XT7 (Noldus, Wageningen, The Netherlands) detection system was used and the videos were saved for later behavioral analysis. A complete suppression of spontaneous locomotion and movements was counted as freezing behavior.

Morris Water Maze

Morris Water Maze with automatic tracking system was employed for assessing the spatial learning and memory of adult mice at the age of 8 to 12 weeks, as described before [1]. All the experimental procedures were performed within the light cycle (9 a.m. to 13 p.m.). The mice were tested in three blocks of training in 9 consecutive days: visible platform training, hidden platform training and probe test. In trainings with platform, the mice were allowed to swim to reach the platform for a maximum period of 1 minute. Otherwise they were guided to the platform by the experimenter. In the visible platform test, no external cue was applied. Mice were trained to reach the flag-cued visible platform for 2 days with 4 trials per day. The platform location and starting position varied for each trial. In the hidden platform training, external cues were hanged onto the wall of the room and the platform was immersed into the opaque water with white non-toxic painting dye. Mice were trained for 6 days with 4

trials per day and the starting position was in a pseudorandom manner. Probe test was performed 1 day after the last hidden platform training. During probe trials, experimental mice were allowed to swim freely for 1 minute without platform placing inside the pool but with same distal cues placed in hidden platform trainings. The starting position is at the opposite side of the previous hidden platform location. Escape latency to the platform was calculated as an evaluation of performance of the mice to locate the target. The performance in probe trial was expressed as time spent in the target quadrant with platform location during hidden platform training. Swimming velocity in each trial was also recorded. The value was calculated by EthoVision™ professional version (EthoVision, Nevertheland).

Immunocytochemistry

Fluorescent staining After being rinsed with 1× PBS, sections were pretreated with blocking solution for 30 minutes at room temperature. Sections were incubated with primary antibodies in diluent at 4°C, for overnight. After washing with 1×PBS for 10 minutes 3 times, sections were incubated with secondary antibodies in PBS at room temperature, for 1 hour. Coverslips were mounted on the section with fluorescence mounting medium (DAKO).

3,3′-Diaminobenzidine (DAB) staining After being rinsed with 1× PBS, sections were quenched in 0.3% H_2O_2 at room temperature, for 30 minutes. Sections were blocked and incubated with the primary antibody at 4°C overnight, and, after washing, exposed to biotinylated secondary antibodies at room temperature, for 2 hours. Immunoreactivity of samples using biotinylated secondary antibodies was detected with VECTASTAIN Elite ABC kit (Vector laboratories, Inc.).

Extra steps for BrdU staining Antigen retrieval was achieved by boiling sections in 10mM citrate buffer at pH 6.0 for 30 minutes. Before blocking, sections were exposed to 2N HCl for 30 minutes for deoxyribonucleic acid hydrolysis, rinsed with PBS and immediately neutralized in boric acid for 15 minutes.

Antibodies BrdU (Abcam, Ab6326, 1:1000), DCX (CHEMICON, AB5910, 1:500), NeuN (CHEMICON, MAB377, 1:1000), GFAP (DAKO, Z0334, 1:5000), pCREB (Cell Signaling, #9198, 1:1000), Ki67 (Abcam, Ab15580, 1:1000)

Western blot analysis

After the behavioral test, mice were decapitated immediately. Hippocampi were collected and stored in -80°C until further process. Hippocampus was collected and lysed in RIPA buffer (150mM NaCl, 1.0% NP-40, 0.5% sodium deoxycholate, 0.1% SDS, 50mM Tris-HCl pH 8.0, EDTA-free protease inhibitors, Roche) for Western blot analysis. Homogenate was centrifuged at 12,000 rpm at 4°C for 15 minutes. Supernatant was transferred to a new tube and protein concentration was determined by BioRad protein assay. The volumes of the sample used were adjusted to give equal loadings to each lane. Proteins were separated by SDS-PAGE gel and transferred onto a polyvinylidene difluoride membrane. The membrane was blocked for 1 hour with 5% non-fat dry milk powder (BioRad) in TBST buffer to eliminate non-specific binding sites. The membrane was then incubated with primary antibody in blocking solution for overnight at 4°C. Detection was performed with peroxidase-conjugated goat anti-mouse or goat anti-rabbit antibody in 1:2500 dilution. Peroxidase activity was revealed using the enhanced chemiluminescence (ECL) method. Image J was used for densitometric analysis of blots. Antibodies used in Western blot included: Epac1 and Epac2 (Kind gifts from Professor J. Bos, 1:2000), PKA (Abcam,

Ab65013, 1:2000), phospho-PKA (Millipore, ABT58, 1:1000), BDNF (Santa Cruz, Sc-546, 1:500), α-tubulin (Sigma, T9026, 1:10000).

Corticosterone measurement

To determine the activity of hypothalamic-pituitary-adrenal (HPA) axis in different lines of mice, corticosterone was measured. Mice were divided into two groups, home caged (resting) or stressed. Blood sample from tail tip were collected between 9:30 to 11:30 A.M. For home caged mice, blood was collected rapidly without disturbance before mice removed from their cages. For stressed mice, blood samples were collected immediately after 30 minutes restraint. Serum was separated by centrifugation and stored at -20°C until assay. Corticosterone levels were measured using the corticosterone ELISA kit (Assay Designs) following the manufacturer's instructions.

Neurotransmitters measurement by LC-MS/MS

Briefly, the chromatographic separation was performed on an Agilent UHPLC 1290 series system (Agilent, Waldbronn, Germany). The mobile phase A consisted of 0.1% formic acid in water ant the mobile phase B consisted of 0.1% formic acid in acetonitrile. The column used was ACE C18 column (3.0 μm i.d., 100 mm × 2.1 mm). A pre-equilibration period of 4 minutes was used between each run. The initial mobile phase composition was isocratic gradient 1.0% (B) in 2 minutes. At 2 minutes, the linear gradient was ramped from 1.0 to 90.0% (B) for 4 minutes and isocratic gradient 90.0% (B) was hold until 10 minutes. The flow rate was 0.2 mL/min and the column temperature was 25°C. The injection volume was 5μL. The running conditions were optimized as below described: drying gas, nitrogen (10

L/min, 325 °C); capillary voltage, 1950 V; scan mode, SRM. The compounds of interest were detected by MS/MS using Agilent QQQ-MS/MS (6410B) equipped with a positive ESI ion source. The detected ion pairs, the acquired fragmentor and the collision energy were tuned using Agilent optimization software (version B02.01). The mass spectrometry calibration was performed using the ESI-L low concentration tuning mix supplied with the apparatus, with aid of Agilent Mass Hunter Chemstation software (version B01.03). Agilent Mass Hunter workstation software (version B.01.00) was used for data acquisition and processing [6].

Reference:

1. Zhang X, Yeung PK, McAlonan GM, Chung SS, Chung SK. Transgenic mice over-expressing endothelial endothelin-1 show cognitive deficit with blood-brain barrier breakdown after transient ischemia with long-term reperfusion. *Neurobiol Learn Mem* 2013; **101:** 46-54.

2. Petit-Demouliere B, Chenu F, Bourin M. Forced swimming test in mice: a review of antidepressant activity. *Psychopharmacology (Berl)* 2005; **177**(3)**:** 245-255.

3. Snyder JS, Soumier A, Brewer M, Pickel J, Cameron HA. Adult hippocampal neurogenesis buffers stress responses and depressive behaviour. *Nature* 2011; **476**(7361)**:** 458-461.

4. Jud C, Schmutz I, Hampp G, Oster H, Albrecht U. A guideline for analyzing circadian wheel-running behavior in rodents under different lighting conditions. *Biological procedures online* 2005; **7:** 101-116.

5. Ding AY, Li Q, Zhou IY, Ma SJ, Tong G, McAlonan GM *et al.* MR diffusion tensor imaging detects rapid microstructural changes in amygdala and hippocampus following fear conditioning in mice. *PLoS One* 2013; **8**(1)**:** e51704.

6. Zhu KY, Fu Q, Leung KW, Wong ZCF, Choi RCY, Tsim KWK. The establishment of a sensitive method in determining different neurotransmitters simultaneously in rat brains by using liquid chromatography–electrospray tandem mass spectrometry. *Journal of Chromatography B* 2011; **879**(11-12)**:** 737-742.
