# Supplementary material for: Anxiety and depression with neurogenesis defects in exchange protein directly activated by cAMP 2-deficient mice are ameliorated by a selective serotonin reuptake inhibitor, Prozac
Source: Transl Psychiatry. 2016 Sep 6;6(9):e881–. doi: 10.1038/tp.2016.129 (PMC5048194; doi:10.1038/tp.2016.129)
Supplement: Supplementary Figures [file tp2016129x2.pdf]

Supplementary Figure 1

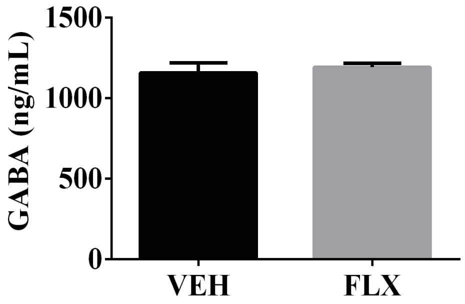

LC-MS/MS measurement of GABA in the hippocampus of vehicle- and fluoxetine-treated Epac2$^{-/-}$ mice. Hippocampi were dissected, homogenized and GABA (γ-aminobutyric acid) was measured by HPLC-MS/MS. No significant difference was detected by unpaired t test. (N=5-6 per group)

Supplementary Figure 2

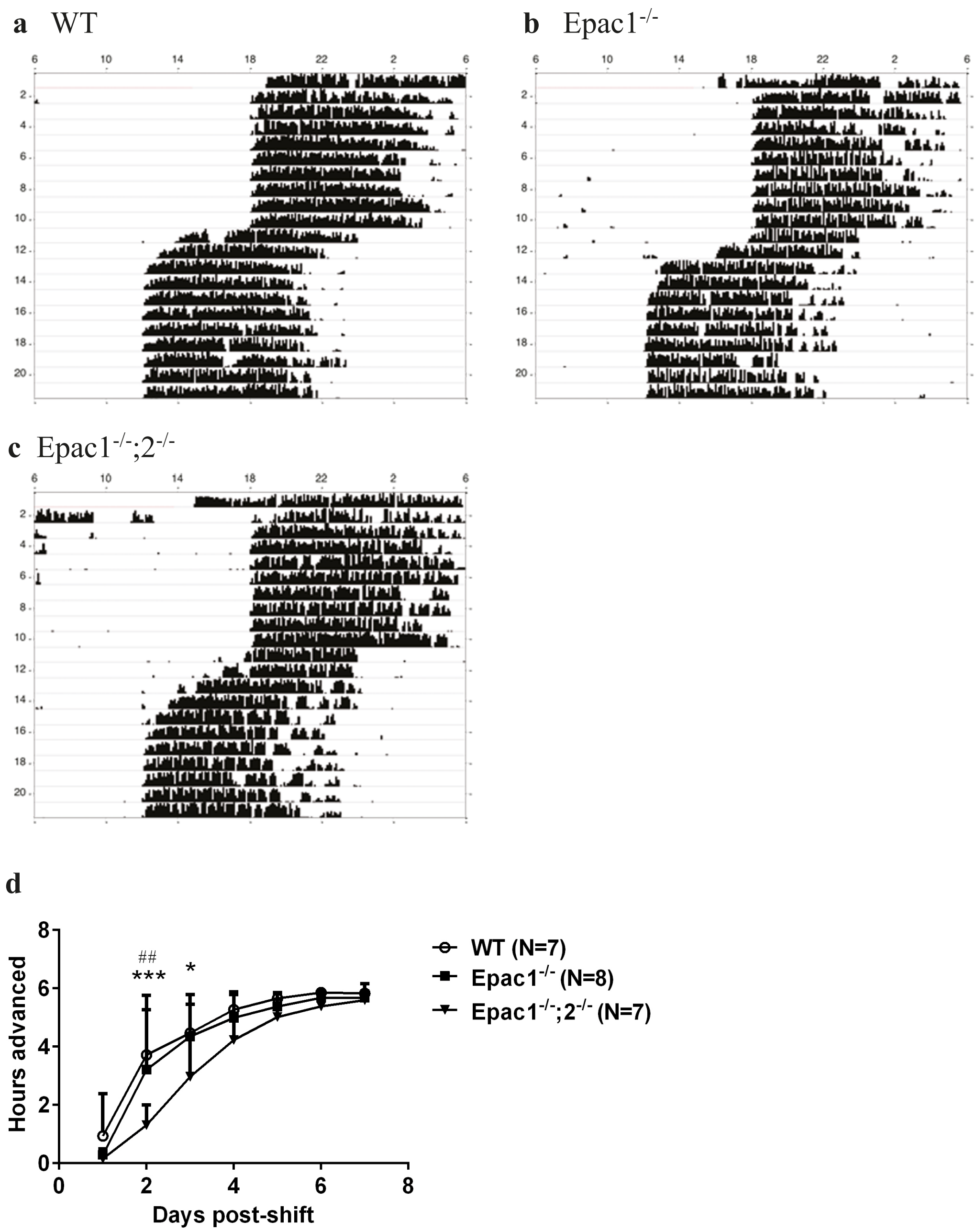

Wheel running activity showing the circadian rhythm of WT, Epac1$^{-/-}$ and Epac1$^{-/-}$;2$^{-/-}$ mice under the light dark cycle shift experiment. In the representative actograms (a-c), each horizontal line indicated one day. Black vertical bars plotted side-by-side represented the activity of the mice, indicated by the number of wheel revolutions in this experiment. The light dark cycle was shift from 18:00-06:00 to 12:00-24:00 on day 11. Synchronization of the internal circadian clock to the environment light-dark signal (re-entrainment) happened several days later. (d) Advance of activity onset after light dark cycle shift was plotted in marked lines (*P<0.05 and ***P<0.001 showing difference between WT and Epac1$^{-/-}$;2$^{-/-}$ mice; ##P<0.01 showing difference between Epac1$^{-/-}$ and Epac1$^{-/-}$;2$^{-/-}$ mice by Tukey's multiple comparison test. N=7-8).

Supplementary Figure 3

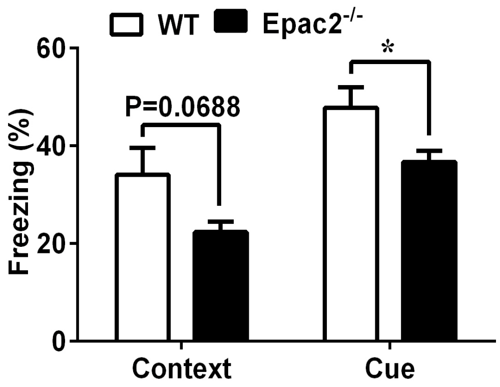

Epac2$^{-/-}$ mice showed moderately impaired cued and contextual fear memory one week after training. Percentages of the freezing behavior were again determined during contextual/cued tests 7/8 days after training, respectively (P=0.0688 for contextual test and P<0.05 for cued test between WT and Epac2$^{-/-}$ mice by unpaired T-test. N=8-9).
